# GLP-1 Receptor Agonists and Suicide Risk in Adults With Chronic Pain: A New User Cohort Across Multi-System EHR data

**DOI:** 10.1192/j.eurpsy.2026.11980

**Published:** 2026-07-31

**Authors:** A. S. Verma, V. Sharma, A. Pathak, R. Gupta

**Affiliations:** 1 Psychiatry, Case Western Reserve University/ Metrohealth, Cleveland, United States; 2 Psychiatry, Dayanand Medical College & Hospital, Ludhiana, India; 3 Psychiatry, Cleveland Clinic Main Campus, Cleveland; 4 Psychiatry, Brigham & Women’s Faulkner Hospital; 5 Psychiatry, Harvard Medical School, Boston, United States

## Abstract

**Introduction:**

Suicidality is a significant concern in patients living with chronic pain, with suicidal ideation reported in 18-50% of this population, and 5-14% attempting suicide. GLP-1 receptor agonists (GLP-1RAs) reduce pain hypersensitivity by acting on peripheral and central nervous systems through glucagon-like peptide-1 (GLP-1) receptors. GLP-1R–based anti-obesity/diabetes therapies, can potentially be useful in high-risk groups such as those with chronic pain, a research gap we aim to fulfill through this cohort study.

**Objectives:**

To compare 12-month risk of suicidality and related psychiatric/health-services outcomes among chronic-pain patients initiating GLP-1 therapies versus other anti-obesity medications (AOMs).

**Methods:**

Retrospective cohort using TriNetX US Collaborative Network (72 HCOs). Adults (≥18) meeting a prespecified chronic-pain phenotype (ICD-10: G89.2x, G89.4, M79.7, chronic headache subtypes, vertebrogenic/other low-back pain, osteoarthritis subtypes) formed the base population. Exposure: GLP-1 agents (semaglutide, liraglutide, dulaglutide, exenatide, lixisenatide, tirzepatide); Comparator: non-GLP AOMs (orlistat, phentermine, bupropion, naltrexone, topiramate). Cohorts mutually excluded the other drug class. Index = first qualifying medication after pain documentation. Outcomes (12-month window, excluding prior outcome): suicidal ideation (R45.851), suicide attempt (T14.91*), any ED visit (CPT 99281–99285), and exploratory psychiatric outcomes (F32.9 depression, F41.9 anxiety, G47.00 insomnia, F11.20 opioid dependence). Propensity-score matching (1:1) balanced age, sex, ethnicity, type-2 diabetes, and BMI percentile.

**Results:**

After matching (N=501,736 per arm) (Table 1), GLP-1 initiation was associated with lower risk of:

Suicidal ideation: RR 0.19 (95% CI 0.17–0.20); HR 0.21 (0.19–0.22).

Suicide attempt: RR 0.13 (0.10–0.17); HR 0.15 (0.11–0.19).

Any ED visit: RR 0.77 (0.76–0.79); HR 0.86 (0.84–0.88). Exploratory outcomes were concordant: depression RR 0.18, anxiety RR 0.42, insomnia RR 0.48, opioid dependence RR 0.25 (all p<0.001) (Table 2).

**Image 1:**
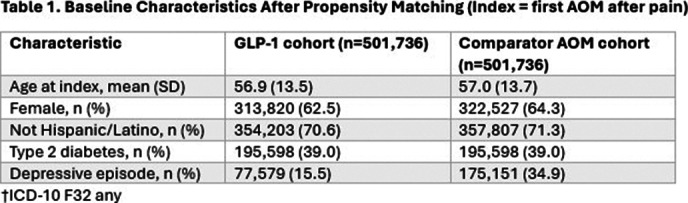
Image 1: Long description.

**Image 2:**
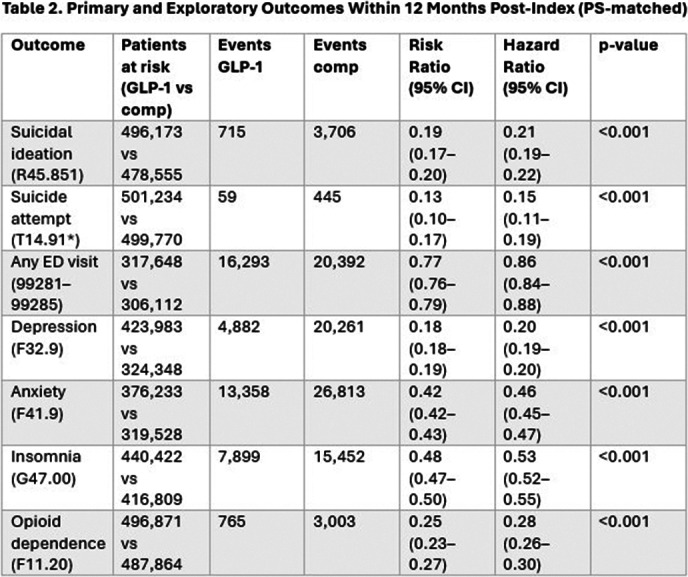
Image 2: Long description.

**Conclusions:**

In adults with chronic pain, GLP-1 therapy versus other AOMs was associated with substantially reduced 12-month suicidality and reduced acute care utilization, with consistent reductions across psychiatric endpoints. These findings are hypothesis-generating and require confirmation with additional confounding control (e.g., baseline psychiatric burden), sensitivity analyses (new-user washout, early-risk windows), and prospective designs.

**Disclosure of Interest:**

None Declared

## Long descriptions

### Image 1: Long description

The table contains three columns: Characteristic, G L P-1 cohort (n=501,736), and Comparator A O M cohort (n=501,736).

* Age at index, mean (S D): G L P-1 cohort is 56.9 (13.5); Comparator is 57.0 (13.7).

* Female, n (%): G L P-1 cohort is 313,820 (62.5); Comparator is 322,527 (64.3).

* Not Hispanic/Latino, n (%): G L P-1 cohort is 354,203 (70.6); Comparator is 357,807 (71.3).

* Type 2 diabetes, n (%): G L P-1 cohort is 195,598 (39.0); Comparator is 195,598 (39.0).

* Depressive episode, n (%): G L P-1 cohort is 77,579 (15.5); Comparator is 175,151 (34.9).

A footnote at the bottom specifies dagger I C D-10 F32 any.

Navigate back to Image 1:.

### Image 2: Long description

The table contains seven outcome rows and six data columns. The columns are: Outcome, Patients at risk (G L P-1 vs comp), Events G L P-1, Events comp, Risk Ratio (95% C I), Hazard Ratio (95% C I), and p-value.

* Suicidal ideation (R45.851): 496,173 vs 478,555 patients; 715 vs 3,706 events; Risk Ratio 0.19 (0.17–0.20); Hazard Ratio 0.21 (0.19–0.22); p-value <0.001.

* Suicide attempt (T14.91*): 501,234 vs 499,770 patients; 59 vs 445 events; Risk Ratio 0.13 (0.10–0.17); Hazard Ratio 0.15 (0.11–0.19); p-value <0.001.

* Any E D visit (99281–99285): 317,648 vs 306,112 patients; 16,293 vs 20,392 events; Risk Ratio 0.77 (0.76–0.79); Hazard Ratio 0.86 (0.84–0.88); p-value <0.001.

* Depression (F32.9): 423,983 vs 324,348 patients; 4,882 vs 20,261 events; Risk Ratio 0.18 (0.18–0.19); Hazard Ratio 0.20 (0.19–0.20); p-value <0.001.

* Anxiety (F41.9): 376,233 vs 319,528 patients; 13,358 vs 26,813 events; Risk Ratio 0.42 (0.42–0.43); Hazard Ratio 0.46 (0.45–0.47); p-value <0.001.

* Insomnia (G47.00): 440,422 vs 416,809 patients; 7,899 vs 15,452 events; Risk Ratio 0.48 (0.47–0.50); Hazard Ratio 0.53 (0.52–0.55); p-value <0.001.

* Opioid dependence (F11.20): 496,871 vs 487,864 patients; 765 vs 3,003 events; Risk Ratio 0.25 (0.23–0.27); Hazard Ratio 0.28 (0.26–0.30); p-value <0.001.

Navigate back to Image 2:.

